# Effect of COVID-19 on patient access to health services for noncommunicable diseases in Latin America: a perspective from patient advocacy organizations

**DOI:** 10.1186/s12939-022-01648-x

**Published:** 2022-04-02

**Authors:** Meredith H. Kruse, Alessandra Durstine, Dabney P. Evans

**Affiliations:** 1 Catalyst Consulting Group, LLC, 1 Bond Street, New York, NY 10012 USA; 2 Hubert Department of Global Health, Rollins School of Public Health, Emory University, 1518 Clifton Road, Atlanta, GA 30322 USA

**Keywords:** Noncommunicable diseases, Health services, Access, Equity, Latin America, COVID-19

## Abstract

**Background:**

The COVID-19 pandemic has been felt acutely in Latin America with several countries having among the highest numbers of SARS-CoV-2 cases and related deaths. Individuals living with underlying health conditions have an increased risk of severe disease or death from COVID-19. Patient advocacy organizations often provide supportive services to these individuals and can offer a unique perspective of the patient experience. The objective of this study was to assess the effects of COVID-19 on access to health services in Latin America, as reported by patient advocacy organizations representing individuals living with autoimmune, chronic, and noncommunicable diseases.

**Methods:**

A cross-sectional study was conducted in August 2020 with patient advocacy organizations in Latin America to measure perceived effects from COVID-19 and reported access to health services among individuals living with autoimmune, chronic, and noncommunicable diseases. An original, online survey was developed and deployed in Spanish and Portuguese. Univariate and bivariate analysis was conducted across two main subject areas: perceived patient effects from COVID-19 and patient access to health services. The main outcomes of analysis considered patient access to care during COVID-19 based on type of chronic illness and geographical region in Latin America.

**Results:**

A total of 81 survey responses were analyzed. A majority (83%) of patient advocacy organizations reported their patients experienced delays receiving their treatment and care services; 52% experienced delays of 30 days or more. Telemedicine was considered available, but not accessible to patients (37%) and a majority (76%) of patients faced challenges with electronic prescriptions. Patients were not likely to receive a multi-month prescription from their doctor (38%) or successfully fill it at the pharmacy (26%).

**Conclusions:**

According to responses from patient advocacy organizations, individuals living with noncommunicable diseases in Latin America have faced unique challenges during the COVID-19 pandemic. As countries re-evaluate their health systems, it is critical that chronic diseases are considered so that all can fully realize the right to health.

**Supplementary Information:**

The online version contains supplementary material available at 10.1186/s12939-022-01648-x.

## Background

On March 11, 2020, the World Health Organization (WHO) Director-General, Dr. Tedros Adhanom Ghebreyesus, officially characterized the 2019 novel coronavirus disease (COVID-19) as a pandemic [1]. Nearly 2 years later, COVID-19 has been reported in 219 countries and territories, accounting for over 410.6 million cases and 5.8 million deaths, with both cases and deaths continuing to rise [2]. The burden of COVID-19 has been acutely felt in Latin America with Argentina, Brazil, Colombia, Mexico, and Peru representing countries with some of the highest numbers of confirmed COVID-19 cases and related deaths [2].

The enormous impact of the COVID-19 pandemic has disrupted the day to day life of populations, economies, and health systems. Countries are faced with the challenge of balancing the priorities of infection prevention, control, and treatment, while also ensuring that essential public health services continue to be safe and accessible. In Latin America, where public health systems are often overburdened, fragmented, and unequal [3], the COVID-19 pandemic has highlighted the failures of underfunding and exacerbated racial, gender, economic, and health disparities [4, 5].

Social determinants of health directly relate to health inequalities [6]. Indigenous people (10%) and people of African descent (21%) represent nearly a third of the population in Latin America and are more likely to live in poorer socioeconomic conditions with limited or no access to healthcare [7], contributing to negative health outcomes, especially during the COVID-19 pandemic. Women account for the majority (72.8%) of workers in healthcare settings in Latin America and have also taken on greater responsibilities at home to care for children during school closures due to COVID-19 [7]; therefore, potential work exposures may increase the risk of virus spread in the home and community. Informal and low wage workers have also felt the brunt of countries’ lockdown measures that have negatively impacted economies; these workers often have limited savings and little to no access to government subsidies [7].

Contributing to these inequities and structural barriers is the risk of severe disease or death from COVID-19 due to underlying health conditions, including noncommunicable diseases (NCDs) like cancers, cardiovascular disease (CVD), and diabetes [8]. In the Region of the Americas, NCDs are the leading cause of death, representing 81%, or 5.8 million, deaths [9]. Traditionally, NCDs and their risk factors have been described as consequences of aging populations, increased wealth, and urbanization that can be prevented through behavior change; in reality, NCDs carry a higher burden of morbidity and mortality among populations living in poverty without access to equitable health systems and do not discriminate by race, gender, or age [10]. The Lancet NCDI (noncommunicable disease and injury) Poverty Commission [10] refers to NCDIs as “under-recognized and poorly-understood” factors in the morbidity and mortality of low income and impoverished populations. NCDIs represent more than a third of the burden of disease among the poorest populations, accounting for approximately 800,000 annual deaths of those 40 years of age and younger [10].

Acknowledging the impact of NCDs on population health, in 2011 the United Nations High Level Meeting on NCDs and its Political Declaration [11] spearheaded discussion and commitments of NCD response that led to the development of Global [12] and Regional [13] Action Plans for the Prevention and Control of NCDs and the inclusion of NCDs in the 2030 Agenda for Sustainable Development [14]. While progress has been made, the plans do not adequately address challenges faced by the poorest of populations impacted by NCDIs and renewed commitments and strategies are needed to prioritize this population. In September 2020, the InterAmerican Task Force on NCDs [15] pledged to strengthen policies and interventions to promote the prevention and control of NCDs and their risk factors among all ages and populations, particularly in the wake of COVID-19 and recovery efforts.

The double burden of NCDs and COVID-19 and the urgent need to address these dueling public health emergencies has begun to gain momentum [16, 17]. In May 2020, the Pan American Health Organization (PAHO) conducted a rapid assessment of NCD service delivery in the Region of the Americas during COVID-19 [18] and reported that many NCD services have been disrupted by varying levels. Member States in the Region reported the types of NCD services disrupted during COVID-19 [18]. Diabetes and hypertension management were most commonly reported as services partially disrupted, while rehabilitation, palliative care, and urgent dental care services were more likely to be reported as completely disrupted [18]. In addition to service disruption, 43% (12/28) reported public health screening programs for NCDs were also postponed [18]. These service interruptions and the delay of critical activities used for the prevention and detection of NCDs can lead to increases in morbidity and mortality during the COVID-19 pandemic and in the future [17].

The negative effects of COVID-19 have been disproportionately experienced within Latin America [4, 5, 7], yet little is known about precisely how COVID-19 has affected access to health care services for individuals living with NCDs. Patient advocacy organizations are a critical point of contact for patients and caregivers, as many provide services, information, support, advocacy, and offer unique insight to the patient experience. The objective of this research was to assess the effects of COVID-19 on patient access to health services in Latin America as reported by patient advocacy organizations who act in the interests of individuals living with autoimmune, chronic, and noncommunicable diseases.

## Methods

A cross-sectional study was conducted in August 2020 with patient advocacy organizations in Latin America to measure perceived effects from COVID-19 and reported access to health services among individuals living with autoimmune, chronic, and noncommunicable diseases.

An original, online survey instrument was developed that included 22 quantitative and qualitative questions (Additional File 1). This manuscript presents results from 12 quantitative questions that asked participants to select one response or yes/no/I do not know. The survey was organized into the following sections: demographics, general impact of COVID-19, patient services, and patient access to care. For the section related to patient access to care, participants were asked to respond based on the experiences reported by the majority of their patients. The survey was written in English, then translated by native speakers into Spanish and Portuguese. The survey instrument was then programmed into the Survey Monkey online platform.

The target population was patient advocacy organizations in Latin America serving individuals living with NCDs. Patient advocacy organizations who were previously registered in the network database of the United Patients Online Academy, a free digital training platform for leaders of non-profit health organizations, were purposively sampled to participate based on their representation of autoimmune, chronic, and noncommunicable diseases and geographical location in Latin America. This included organizations from Argentina, Bolivia, Brazil, Chile, Colombia, Costa Rica, Cuba, Dominican Republic, Ecuador, El Salvador, Guatemala, Honduras, Mexico, Nicaragua, Panama, Paraguay, Peru, Suriname, Uruguay, and Venezuela. Within the organizations, the eligible participants included individuals with familiarity of the strategic priorities of the organization, patient services provided, and reported experiences from patients served, specifically during the COVID-19 pandemic.

The online survey was launched August 5th and closed August 31st, 2020. Each identified organization was sent an email invitation from the United Patients Online Academy, which included a brief explanation of the purpose of the research and a URL link to the online survey. The survey link was also shared on United Patients Online Academy’s social media networks. Due to the distribution methods, it was not possible to determine the number of people who received the invitation to participate. By following the survey link, individuals were provided a statement of research on the title page of the survey and were asked if they were interested in participating. If the individual selected “no,” the survey concluded. If the individual selected “yes,” the following page of the survey described the survey format and eligibility requirements, provided contact information for the Principal Investigators, and included all elements of informed consent prior to the commencement of the survey. If consent was not provided, the survey concluded.

Data cleaning was conducted for data quality assurance to prevent duplication and ensure unique user and complete responses. Univariate and bivariate analysis was conducted across two main subject areas: perceived patient effects from COVID-19 and reported patient access to health services. The perceived effects of the COVID-19 pandemic on patients were categorized through analysis as personal, structural, and clinical factors. The main outcomes of analysis considered patient access to care during COVID-19 based on type of chronic illness or disease and geographical region in Latin America. The findings represent the aggregated, unweighted results of the patient access to care portion of the survey.

The study was reviewed by the Emory University Institutional Review Board in Atlanta, GA, U.S.A. and found to be exempt from review since it was classified as public health practice.

## Results

A total of 134 surveys were submitted. Responses were reviewed based on confirmation of informed consent and completeness (defined as responding to at least one of the 6 key access to care questions); 53 responses were excluded from analysis due to the respondent not meeting the aforementioned requirements. A remaining sample of 81 patient advocacy organizations (*N* = 81) were included in analysis.

The participating patient advocacy organizations represented 18 countries in Latin America, including the Andean Region (27%), Central America and Cuba (23%), and Mexico (22%) (Table 1). Nine disease areas were represented by the organizations with the majority categorized as cancer (37%), Multiple Sclerosis (21%), and other chronic and noncommunicable diseases (16%). Most (53%) participants held executive leadership roles within their organizations.

**Table 1:** Demographics of Patient Advocacy Organizations Surveyed in Latin America, 2020, N = 81

	n	%
**Country**		
Argentina	6	7.41
Brazil	11	13.58
Chile	2	2.47
Colombia	11	13.58
Costa Rica	3	3.70
Cuba	2	2.47
Dominican Republic	3	3.70
Ecuador	1	1.23
El Salvador	5	6.17
Guatemala	1	1.23
Honduras	3	3.70
Mexico	18	22.22
Nicaragua	1	1.23
Panama	1	1.23
Paraguay	1	1.23
Peru	7	8.64
Uruguay	2	2.47
Venezuela	3	3.70
**Region**		
Andean Region	22	27.16
Brazil	11	13.58
Central America and Cuba	19	23.46
Mexico	18	22.22
Southern Cone	11	13.58
**Disease**		
Cancer	30	37.04
Cardiovascular Disease	2	2.47
Diabetes	5	6.17
Lupus	3	3.70
Multiple Sclerosis	17	20.99
Patient Support (General)	3	3.70
Rare Disease	9	11.11
Rheumatoid Arthritis	4	4.94
Other	8	9.88
**Disease Category**		
Autoimmune	12	14.81
Cancer	30	37.04
Multiple Sclerosis	17	20.99
Rare Disease	9	11.11
Other chronic and NCDs	13	16.05
**Role**		
Executive Leadership	43	53.09
Programs and/or Patient Services	15	18.52
Volunteer	9	11.11
Advocacy	5	6.17
Fundraising/Partnership Development	4	4.94
Administration/Finances	1	1.23
Other	4	4.94

### Effects of COVID-19

According to patient advocacy organizations, the COVID-10 pandemic effected patients in many ways. A strong majority (83%) stated their patients reported delays in receiving their treatment and care services, defined as all forms of medical treatment, such as screening services, regular check-ups, in-hospital treatment protocols, surgery, clinical trials, among others (Table 2). Of those who reported delays, 63%, or 42 of 67, organizations had a majority of patients who experienced a delay of more than 30 days.

**Table 2:** Effects of COVID-19 on Patients According to Patient Advocacy Organizations in Latin America, 2020, N = 81

	Yes	No	Do not Know	Missing
	n (%)	n (%)	n (%)	n
In general, have the majority of your patients reported experiencing delays in receiving treatment/care?				
**Patient Reported Delays**				
Treatment and care delays	67 (82.72)	13 (16.05)	NA	1
Treatment and care delays > 30 Days	42 (51.85)	38 (46.91)	NA	1
Treatment and care delays ≤ 30 Days	25 (30.86)	55 (67.90)	NA	1
What are some of the biggest impacts the COVID-19 pandemic has had on your patients? Select “yes” if the issue has been impacted, “no” if the issue has not been impacted, or “I do not know.”				
**Personal Factors**				
Overall Impact	NA (90.95)	NA (4.12)	NA (3.29)	NA
Patient fear of going to the hospital for treatment	75 (92.59)	4 (4.94)	2 (2.47)	0
Increased feelings of isolation and depression	74 (91.36)	4 (4.94)	1 (1.23)	2
Financial uncertainty	72 (88.89)	2 (2.47)	5 (6.17)	2
**Structural Factors**				
Overall Impact	NA (63.95)	NA (19.01)	NA (15.56)	NA
Consultations and/or surgeries postponed	74 (91.36)	4 (4.94)	2 (2.47)	1
Delayed screening and late diagnosis	65 (80.25)	10 (12.35)	5 (6.17)	1
Limited or no access to telemedicine	47 (58.02)	20 (24.69)	13 (16.05)	1
Treatment not available due to limited staffing	38 (46.91)	29 (35.80)	12 (14.81)	2
Decreased participation in clinical trials	35 (43.21)	14 (17.28)	31 (38.27)	1
**Clinical Factors**				
Overall Impact	NA (60.08)	NA (30.86)	NA (7.41)	NA
Lapse in treatment	57 (70.37)	19 (23.46)	4 (4.94)	1
Medication not available due to medication stock out	46 (56.79)	28 (34.57)	6 (7.41)	1
Change in treatment protocols	43 (53.09)	28 (34.57)	8 (9.88)	2

Participating patient advocacy organizations reported a near consensus (91%) that personal factors, such as mental health and finances, had the greatest effects on their patients (Table 2). This was followed by structural (64%) factors or issues related to the health system, and clinical (60%) factors that directly related to the patient and their individual health. Patient fear of going to the hospital (93%), patient mental health (91%), health system or clinician postponement of consultations and/or surgeries (91%), patient financial uncertainty (89%), and health system delayed screenings and late diagnoses (80%) were considered the factors with the greatest effects on patients according to the patient advocacy organizations.

At the start of the pandemic, 26% of patient advocacy organizations were contacted by patients and caregivers about access to treatment and care that had been interrupted by COVID-19 (Table 3). The second most common reason for contact was to receive general information about access to treatment and care not related to COVID-19 (25%). Similar reasons were seen in August 2020 (24 and 28%, respectively).

**Table 3:** Patient/Caregiver Reasons for Contact during COVID-19 among Patient Advocacy Organizations in Latin America, 2020

	Start of COVID-19	Today (Aug. 2020)^a^
	n/N (%)	
What was the main reason patients and/or caregivers contacted your organization?		
**Main Reasons for Contact**		
Access to treatment and care that has been interrupted by COVID-19	21/81 (25.93)	19/78 (24.36)
General information about access to treatment and care (not related to COVID-19)	20/81 (24.69)	22/78 (28.21)
General information about treatment and care protocols (not related to COVID-19)	16/81 (19.75)	11/78 (14.10)
Information about COVID-19	8/81 (9.88)	4/78 (5.13)
Psychosocial support	5/81 (6.17)	8/78 (10.26)
Patients' rights	4/81 (4.94)	6/78 (7.69)
Financial support	2/81 (2.47)	5/78 (6.41)
Other	5/81 (6.17)	3/78 (3.85)

^a^Missing not included in denominator

### Patient access to health services

Evidence-based mitigation strategies that were recommended to ensure continuity of care and limit potential exposure to COVID-19 infection include telemedicine, electronic prescriptions, and multi-month scripting and dispensing [19]. The use of digital platforms, such as telemedicine, which allows for virtual provider consultations, and the integration of electronic prescriptions that can be virtually prescribed and processed by the pharmacy, are key approaches for comprehensive essential health service delivery [19]. Likewise, multi-month scripting and dispensing (MMSD) that supports differentiated care and patient-centered service delivery, enables patients to receive longer prescriptions of 90 days or more so they do not need to return to clinic for a new prescription or return to the pharmacy for monthly refills [19, 20]. The availability and how patients accessed these recommended health services during the COVID-19 pandemic varied by disease and geographical region (Table 4).

**Table 4:** Patient Access to Health Services during COVID-19, by Disease Category and Geographical Region, 2020

			Disease Category					Geographical Region			Total
	Auto- immune	Cancer	Multiple Sclerosis	Rare Disease	Other NCDs	Andean Region	Brazil	Central America and Cuba	Mexico	Southern Cone	
						n/N (%)					
**Telemedicine**^**a**^											
Accessed, private	1/12 (8.3)	5/30 (16.7)	2/17 (11.8)	5/9 (55.6)	2/13 (15.4)	1/22 (4.6)	5/11 (45.5)	2/19 (10.5)	2/18 (11.1)	5/11 (45.5)	15/81 (18.5)
Accessed, public	2/12 (16.7)	5/30 (16.7)	4/17 (23.5)	1/9 (11.1)	2/13 (15.4)	8/22 (36.4)	0	2/19 (10.5)	3/18 (16.7)	1/11 (9.1)	14/81 (17.3)
Accessed, private/public	2/12 (16.7)	7/30 (23.3)	1/17 (5.9)	0	1/13 (7.7)	3/22 (13.6)	2/11 (18.2)	4/19 (21.1)	0	2/11 (18.2)	11/81 (13.6)
Not accessible	5/12 (41.7)	10/30 (33.3)	5/17 (29.4)	3/9 (33.3)	7/13 (53.9)	10/22 (45.5)	4/11 (36.4)	4/19 (21.1)	9/18 (50.0)	3/11 (27.3)	30/81 (37.0)
Not available	2/12 (16.7)	3/30 (10.0)	5/17 (29.4)	0	1/13 (7.7)	0	0	7/19 (36.8)	4/18 (22.2)	0	11/81 (13.6)
**Electronic Prescriptions**^**a**^											
Received, filled	2/12 (16.7)	7/30 (23.3)	5/17 (29.4)	4/8(50.0)	1/12 (8.3)	6/21 (28.6)	0	8/18 (44.4)	2/18 (11.1)	3/11 (27.3)	19/79 (24.1)
Received, not filled	3/12 (25.0)	4/30 (13.3)	5/17 (29.4)	3/8 (37.5)	2/12 (16.7)	10/21 (47.6)	1/11 (9.1)	2/18 (11.1)	0	4/11 (36.4)	17/79 (21.5)
Not received	3/12 (25.0)	13/30 (43.3)	2/17 (11.8)	0	6/12 (50.0)	3/21 (14.3)	10/11 (90.9)	1/18 (5.6)	7/18 (38.9)	3/11 (27.3)	24/79 (30.4)
Not available	4/12 (33.3)	6/30 (20.0)	5/17 (29.4)	1/8 (12.5)	3/12 (25.0)	2/21 (9.5)	0	7/18 (38.9)	9/18 (50.0)	1/11 (9.1)	19/79 (24.1)
**Multi-Month Scripting and Dispensing**^**a**^											
Prescribed, filled	7/12 (58.3)	6/30 (20.0)	9/15 (60.0)	2/8 (25.0)	4/12 (33.3)	8/20 (40.0)	4/11 (36.4)	9/17 (52.9)	4/18 (22.2)	3/11 (27.3)	28/77 (36.4)
Prescribed, not filled	2/12 (16.7)	9/30 (30.0)	3/15 (20.0)	2/8 (25.0)	4/12 (33.3)	9/20 (45.0)	2/11 (18.2)	2/17 (11.8)	6/18 (33.3)	1/11 (9.1)	20/77 (26.0)
Not prescribed	3/12 (25.0)	15/30 (50.0)	3/15 (20.0)	4/8(50.0)	4/12 (33.3)	3/20 (15.0)	5/11 (45.5)	6/17 (35.3)	8/18 (44.4)	7/11 (63.6)	29/77 (37.7)
**Palliative Care**^**b**^											
Available	5/8 (62.5)	11/23 (47.8)	9/10 (90.0)	2/4 (50.0)	3/7 (42.9)	7/13 (53.9)	6/7 (85.7)	7/13 (53.9)	4/11 (36.4)	6/8 (75.0)	30/52 (57.7)
Not Available	3/8 (37.5)	12/23 (52.2)	1/10 (10.0)	2/4 (50.0)	4/7 (57.1)	6/13 (46.2)	1/7 (14.3)	6/13 (46.2)	7/11 (63.6)	2/8 (25.0)	22/52 (42.3)

^a^Missing not included in denominator

^b^Not applicable and missing not included in denominator

Half (51%) of patient advocacy organizations reported that telemedicine was not accessible (37%) or available (14%) for the majority of their patients. Patient advocacy organizations that represented individuals living with autoimmune diseases (42%) and cancers (33%) reported that telemedicine was available in their countries, but not accessible. Likewise, Mexico (50%) and the Andean Region (45%) were most likely to not have access to telemedicine. Central America and Cuba (37%) reported telemedicine was not available at all.

A majority (76%) of patient advocacy organizations reported challenges related to receiving (54%) and processing (22%) electronic prescriptions. One third (33%) of autoimmune organizations reported electronic prescriptions were not available for their patients, while 43% of cancer organizations described their patients had not received an electronic prescription from their doctor. Half (50%) of patient advocacy organizations in Mexico reported electronic prescriptions were not available. Brazilian patients did not receive an electronic prescription from their doctors, according to a majority (91%) of the patient advocacy organizations from the country.

Many (64%) patient advocacy organizations reported MMSD was not prescribed (38%) or was prescribed, but not successfully filled by the pharmacy (26%). Cancer (50%) and rare disease (50%) patients were most likely to not be prescribed a multi-month prescription. This was also seen in the Southern Cone (64%), Brazil (45%), and Mexico (44%), with patient advocacy organizations reporting a majority of their patients were not prescribed longer prescriptions. In the Andean Region, nearly half (45%) of organizations reported their patients were prescribed, but the pharmacy was unable to fill the multi-month allotment.

A majority (58%) of patient advocacy organizations reported their eligible patients had access to pain management medication or palliative care. However, most cancer (52%) and rare disease (50%) organizations reported palliative care was not available for their patients. Likewise, in Mexico, a majority (64%) of patient advocacy organizations reported their eligible patients were not able to access palliative care.

## Discussion

The COVID-19 pandemic has presented immense challenges for economies and health systems throughout the world. This is the first study that considers the perspective and expertise of the patient advocacy organizations representing individuals living with autoimmune, chronic, and noncommunicable diseases to provide a snapshot of how patients are accessing essential health services in Latin America during the COVID-19 pandemic. Our results describe the patient experience and suggest there are health access inequities based on disease areas and geographical regions.

All participating patient advocacy organizations recognized the impact of disrupted or delayed health services, such as postponed consultations or surgeries and delayed diagnostic screenings, which may lead to an increased burden on health systems in the future when patients return to care with urgent needs or present at an advanced stage of disease [17]. Many patients reported to the patient advocacy organizations that their own treatment or care was delayed by more than 30 days. These delays related to management of NCDs during COVID-19 and their potential health impacts have been similarly highlighted in the literature [21–25]. Kiss et al. [21] described substantial decreases in rates of admission for acute CVD, suggesting that individuals are not seeking or delaying care, which may have implications for increased disease severity or mortality, and may lead to other complications, such as heart failure [22]. Likewise, the number of cancer diagnoses have decreased because screening and early detection services have been disrupted, treatments have been delayed, and enrollment in clinical trials has become difficult, which all pose a major risk to cancer care throughout the globe [22–24]. Barone et al. [25] also described individuals living with diabetes in South and Central America experienced difficulty accessing health services and a lack of medicines or supplies during the COVID-19 pandemic, which may lead to uncontrolled diabetes and exacerbate chronic complications among individuals who are immunocompromised [22].

In addition to delays or disruptions to services, patient advocacy organizations also reported patient challenges in accessing services that are uniquely posed to support safety and wellness during public health emergencies, including telemedicine, electronic prescriptions, and multi-month scripting and dispensing [19, 20]. While telemedicine was largely available in the countries represented, patients were unable to access services demonstrating a disconnect between policy and implementation, which may be the result of a digital divide or other systematic challenges. Likewise, the ability for doctors to prescribe electronic prescriptions to patients that are then received and filled by pharmacies or clinics is critical; however, many patient advocacy organizations reported their patients were unable to successfully benefit from this system, though available, due to challenges with doctors not prescribing electronically and with the electronic prescriptions not being filled by the pharmacy. These obstacles may be due to prescriber hesitancy or an inability for pharmacies to receive and process the technology. Multi-month scripting and dispensing supports patient-centered service delivery [20] but was not widely implemented. Patient advocacy organizations reported challenges with patients being offered longer prescriptions and/or successfully filling the prescription at the pharmacy or clinic, suggesting public and/or private pharmacy systems are not complying or do not have the supply to fill extended prescriptions. These strategies are not unique to COVID-19; they can and should be adopted in any public health emergency in the future to facilitate access to and continuity of essential health services, as well as mitigate potential exposures for populations at higher risk of adverse or severe health outcomes.

As the COVID-19 pandemic continues, it is critical to examine the challenges faced by individuals living with NCDs as they seek services to manage their disease, while also attempting to avoid infection from a potentially deadly virus. This study gives voice to these patients through the unique perspective of the patient advocacy organizations that represent them. It reveals ways in which the health systems in Latin America are failing some of the most vulnerable. After all, it is the responsibility of governments to strategize ways to strengthen their health systems to mitigate inequities and ensure accessibility of health services for their populations. All 18 of the Latin American countries represented in this study have signed or ratified the International Covenant on Economic, Social and Cultural Rights (ICESCR), which establishes “the right of everyone to the enjoyment of the highest attainable standard of physical and mental health” [26]. General Comment 14 of the ICESCR [27] specifies the State must ensure health systems and services are *available* and *accessible* to the entire population with ethical and cultural *acceptability* and the provision of medically appropriate, *quality* services. The State must also *respect, protect,* and *fulfill* the right to health for its population through its core obligations and *progressive realization* in the “prevention, treatment and control of diseases” [27]. Based on our findings, the countries represented by the participating patient advocacy organizations are falling short of their obligations to guarantee a right to health for their populations living with NCDs.

This study includes limitations, which should be considered in the interpretation of the findings. The study design invited patient advocacy organizations to participate; these organizations are more likely to see and interpret challenges faced by their patients through the lens of an advocate that represents the voice of marginalized groups and not necessarily the majority that public health policy is designed to support. Likewise, those who chose to participate in the study may be more likely to report problems, which can introduce self-selection bias. The participant responses were to reflect the experiences of the majority of patients who contacted the participating patient advocacy organizations, though this method may be prone to bias and cannot be formally validated. The study was conducted in August 2020, months after the first cases of COVID-19 were confirmed in Latin American countries, and as such, the responses reflected a cross-sectional snapshot of the effects of COVID-19 that may have changed from earlier stages in the pandemic and may change again as the pandemic continues. The sample sizes for some disease areas and countries represented by the patient advocacy organizations were small and results should be interpreted accordingly. Also, an accurate response rate cannot be determined, as the survey link was shared through various networks and publicly available.

Studies with individuals living with autoimmune, chronic, and noncommunicable diseases are needed to further assess how they are seeking health services during the COVID-19 pandemic and the barriers they face, as well as with the public and private health systems, including pharmacies, to better understand the systemic and logistical aspects of health service delivery for populations living with comorbidities during public health emergencies.

## Conclusions

The COVID-19 pandemic has exposed the systemic inequities of the world where the poorest and populations at highest risk are the most severely impacted. Health challenges that are exacerbated by racial, gender, and other social determinants of health are only compounded in public health emergencies. According to the responses from patient advocacy organizations in Latin America, individuals living with autoimmune, chronic, and noncommunicable diseases have faced unique access challenges during the COVID-19 pandemic. Our findings showed that patients are experiencing lengthy delays of 30 days or more to access their treatment and care. Health services, such as telemedicine, electronic prescriptions, and multi-month scripting and dispensing, are available within countries but are not accessible by all. As governments re-evaluate their COVID-19 strategies and health systems, it is important to recognize the unique burdens of populations at higher risk and consider population health as a priority for human security. It is critical that governments proactively address inequalities and make investments today in order to build back stronger health systems for tomorrow.

## Supplementary Information

**Additional file 1:.** COVID-19 Survey for PAGs in LATAM. Description: English version of the online survey instrument used.

## Data Availability

The dataset used and analyzed during the current study are available from the corresponding author on reasonable request.

## Abbreviations

COVID-19: 2019 Novel Coronavirus Disease caused by SARS-CoV-2
CVD: Cardiovascular Disease
ICESCR: International Covenant on Economic, Social and Cultural Rights
MMSD: Multi-month Scripting and Dispensing
NCDs: Noncommunicable Diseases
NCDIs: Noncommunicable Diseases and Injuries
PAHO: Pan American Health Organization
WHO: World Health Organization
