# Supplementary material for: Effect of COVID-19 on patient access to health services for noncommunicable diseases in Latin America: a perspective from patient advocacy organizations
Source: Int J Equity Health. 2022 Apr 2;21:45. doi: 10.1186/s12939-022-01648-x (PMC8976438; doi:10.1186/s12939-022-01648-x)
Supplement: Supplementary file 1 — Additional file 1:. COVID-19 Survey for PAGs in LATAM. Description: English version of the online survey instrument used. [file 12939_2022_1648_MOESM1_ESM.pdf]

COVID-19 Survey for Patient Organizations in LATAM:

"The Impact of COVID-19 on Patient Organizations and their Patients in Latin America"

Introduction

Hello! Thank you for your interest in our online research survey. The purpose of this survey is to understand the impacts of the SARS-CoV-2 (COVID-19) pandemic on patient organizations and their patients in Latin America. The results of this survey will help identify realities and barriers faced during COVID-19 that should be addressed to improve lives within the region.

All patient organizations based in Latin America are invited to participate in our confidential online research survey.

1. Would you like to participate in this survey? Yes/No

Thank you for agreeing to participate in this survey, which seeks to understand the impact of SARS-CoV-2 (COVID-19) and identify barriers faced by patient organizations and their patients in Latin America.

The online research survey is divided into 5 sections: demographics, general impact of COVID-19, patient services, patient access to care, and next steps. We anticipate the survey will take approximately 15 minutes to complete. All responses are confidential. Your participation is entirely voluntary and you may withdraw at any point during the survey. There are no direct benefits to participating. However, your participation is greatly appreciated.

Eligibility: All patient organizations based in Latin America are eligible to participate. Participants should be familiar with the strategic priorities of the organization, the patient services provided by the organization, and reported experiences from patients served, specifically during the COVID-19 pandemic.

The Principal Investigators are Alessandra Durstine and Meredith Kruse of United Patients Online Academy. If at any point you have questions or concerns about this survey, please contact us (contacto@unitedpatientsacademy.org).

2. Do you consent to participate in this survey? Yes/No

I. Demographics

In this first section, we would like to ask you some basic questions about your patient organization.

3. Please select the focus of your organization:
    a. Cancer
    b. Cardiovascular disease
    c. Diabetes

    d. Multiple Sclerosis
    e. Organ Transplants
    f. Rare Disease
    g. Rheumatoid Arthritis
    h. Other: describe (open response)

4. Please select the country in which your organization is based:
    a. Argentina
    b. Bolivia
    c. Brazil
    d. Chile
    e. Colombia
    f. Costa Rica
    g. Cuba
    h. Dominican Republic
    i. Ecuador
    j. El Salvador
    k. Guatemala
    l. Honduras
    m. Mexico
    n. Nicaragua
    o. Panama
    p. Paraguay
    q. Peru
    r. Suriname
    s. Uruguay
    t. Venezuela

5. Please select your primary role in your organization:
    a. Executive Leadership
    b. Administration/Finances
    c. Fundraising/Partnership Development
    d. Programs and/or Patient Services
    e. Communications and Media
    f. Advocacy
    g. Research
    h. Volunteer
    i. Other (open response)

II. General Impact of COVID-19

In this next part of the survey, we would like to learn more about the impact COVID-19 has had on your patient organization and the patients you support.

6. On a scale of 0-10, what is the impact of COVID-19 on your organization finances? 0 = little to no impact; 5 = moderate impact; 10 = drastic impact on sustainability of organization

7. Have you changed strategies in this year's annual plan because of COVID-19?
   a. Yes
   b. No

8. If yes, please select which of the following strategies has changed the **most**.
   a. Patient services focused on psychological support
   b. Patient services focused on access to treatment
   c. Patient services focused on caregiver/family support
   d. Advocacy
   e. Research
   f. Other

   Please explain how the strategy has changed: (open response)

9. What are some of the **biggest** impacts the COVID-19 pandemic has had on your patients? Please select "yes" if the issue has been impacted, "no" if the issue has not been impacted, or "I do not know."
   a. Delayed screening and late diagnosis
   b. Consultations and/or surgeries are postponed
   c. Lapse in treatment
   d. Change in treatment protocols
   e. Treatment not available due to limited staffing
   f. Medication not available due to mediation stock out
   g. Limited or no access to telemedicine
   h. Decreased participation in clinical trials
   i. Financial uncertainty
   j. Increased feelings of isolation and depression
   k. Patient fear of going to the hospital for treatment
   l. Other: describe (open response)

III. Patient Services

Here, we would like to understand how the COVID-19 pandemic has impacted the patient services you offer as a patient organization.

10. How has the COVID-19 pandemic impacted demand for the services you offer?
    a. Demand has **significantly** increased and now have need to expand capacity

b. Demand has **moderately** increased with more calls and interactions through email and online networks
c. No difference in demand
d. Demand has **decreased** with less calls and interactions through email and online networks

11. At the beginning of the COVID-19 pandemic, what was the **main** reason patients and/or caregivers contacted your organization?
    a. General information about treatment and care protocols (not related to COVID-19)
    b. General information about access to treatment and care (not related to COVID-19)
    c. Access to treatment and care that has been interrupted by COVID-19
    d. Financial support
    e. Psychosocial support
    f. Patient rights
    g. Information about COVID-19
    h. Other: describe (open response)

12. Today, what is the **main** reason patients and/or caregivers contact your organization?
    a. General information about treatment and care protocols (not related to COVID-19)
    b. General information about access to treatment and care (not related to COVID-19)
    c. Access to treatment and care that has been interrupted by COVID-19
    d. Financial support
    e. Psychosocial support
    f. Patient rights
    g. Information about COVID-19
    h. Other: describe (open response)

13. Do you anticipate reducing services due to COVID-19?
    a. Yes, we have plans to reduce services
    b. No current plans to reduce services

14. Have you increased or created new programs or patient services due to COVID-19?
    a. No, no new programs or services
    b. Yes, we have increased capacity of current programs or services
    c. Yes, new programs or services have been created

    Please describe how programs of services have increased and/or the new programs or services that have been developed: (open response)

IV. Patient Access to Care

In this section, we would like to learn how the COVID-19 pandemic has directly impacted patients' access to treatment and care. Please reflect on experiences reported by the **majority** of your

patients who have interacted with your organization from the start of the pandemic in your country to present day.

15. In general, have the **majority** of your patients reported experiencing delays in receiving treatment/care? Treatment/care includes all forms of medical treatment, such as screening services, regular check-ups, in-hospital treatment protocols, surgery, clinical trials, among others.
    a. Yes, the majority of patients report delays **less than 30 days**
    b. Yes, the majority of patients report delays **more than 30 days**
    c. No, the majority of patients have not reported experiencing delays in receiving treatment/care

16. If your patients have reported experiencing delays in receiving treatment/care, what type of treatment/care has **most** frequently been delayed?
    a. Screening services
    b. Regular check-ups
    c. In-hospital treatment protocols
    d. Surgery
    e. Clinical trials
    f. Other: describe (open response)

17. In general, have the **majority** of your patients who have tried or wanted to access telemedicine been able to do so?
    a. Yes, with a private health plan
    b. Yes, through the public health system
    c. Yes, both with a private health plan and the public health system
    d. No, patients have not been able to access telemedicine
    e. Telemedicine is not available in my country

18. In general, have the **majority** of your patients been able to receive electronic prescriptions from their doctors?
    a. Yes, they received an electronic prescription and successfully retrieved the medicine at the pharmacy
    b. Yes, they received an electronic prescription, but could not retrieve the medicine at the pharmacy
    c. No, the patients have not received electronic prescriptions from their doctors
    d. Electronic prescriptions are not available in my country

19. In general, have the **majority** of your patients been able to receive multi-month (90-day) prescriptions at the pharmacy or health post?
    a. Yes, the doctor prescribed a 90-day medication and they received it at the pharmacy or health post
    b. The doctor prescribed the 90-day medication, but the pharmacy or health post could not complete the 90-day prescription
    c. No, multi-month prescriptions were not prescribed by the doctor

20. In general, have the **majority** of your eligible patients been able to access pain management medication?
    a. Yes
    b. No
    c. Not applicable

21. In your country, are there national or local guidelines relating to treatment and care during COVID-19?
    a. Yes
    b. No
    c. I do not know

    Please describe your opinion about the national or local guidelines in your country: (open response)

V. Next Steps

Our final section includes a few questions seeking additional feedback or comment.

22. Do you have any other thoughts or comments to share about how COVID-19 has impacted your organization and/or patients? (open response)

Conclusion: This now concludes the survey. Thank you for your participation in our research, "The Impact of COVID-19 on Patient Organizations and their Patients in Latin America."

Disqualification: Thank you for your interest in our survey. You have decided to not participate. This now concludes the survey.
